# Supplementary material for: Unique transcriptomic landscapes identified in idiopathic spontaneous and infection related preterm births compared to normal term births
Source: PLoS One. 2019 Nov 8;14(11):e0225062. doi: 10.1371/journal.pone.0225062 (PMC6839872; doi:10.1371/journal.pone.0225062)
Supplement: S1 Table — (DOCX) [file pone.0225062.s001.docx]

**S1 Table: Power calculations for number of transcriptomes needed for study**

| **Symbol** | **Description** | **90% power** | **80% power** | **70% power** |
| --- | --- | --- | --- | --- |
| **Alpha (a)** | Type 1 Error | 0.05 | 0.05 | 0.05 |
| **Beta (b)** | Power | 0.9 | 0.8 | 0.7 |
| **Mu (m)** | Counts^1^ | 50 | 50 | 50 |
| **Sigma (s)** | Coefficient of Variation^2^ | 0.43 | 0.43 | 0.43 |
| **Delta (D)** | Fold Change (Effect Size) | 2 | 2 | 2 |
|  | Number of samples | 9 | 7 | 5 |

^1^Counts equal read depth 50 million is sufficient for this experiment based on previous data

^2^Coefficient of Variation = average human variation calculated from (Hart et al. 2013)
