# Supplementary material for: Unique transcriptomic landscapes identified in idiopathic spontaneous and infection related preterm births compared to normal term births
Source: PLoS One. 2019 Nov 8;14(11):e0225062. doi: 10.1371/journal.pone.0225062 (PMC6839872; doi:10.1371/journal.pone.0225062)
Supplement: S2 Table — (DOCX) [file pone.0225062.s002.docx]

**S2 Table: Candidate genes associated isPTB molecular signature**

|  | **isPTB vs AHC** | | **isPTB vs TB** | | **AHC vs TB** | |
| --- | --- | --- | --- | --- | --- | --- |
| **Gene ID** | **Log2**  **Fold Change** | **Adjusted**  **P value*** | **Log2**  **Fold Change** | **Adjusted**  **P value*** | **Log2**  **Fold Change** | **Adjusted**  **P value*** |
| *PAEP* | 5.3 | 1.32E-03 | 7.1 | 1.50E-05 | 1.8 | 3.83E-01 |
| *IGFBP1* | 5.2 | 8.93E-04 | 7.6 | 3.40E-07 | 2.4 | 1.34E-01 |
| *PRL* | 4.3 | 3.45E-04 | 6.3 | 1.18E-07 | 2.0 | 1.30E-01 |
| *CHRDL1* | 4.0 | 1.24E-02 | 6.8 | 1.11E-05 | 2.8 | 1.06E-01 |
| *GNLY* | 3.9 | 1.87E-05 | 3.9 | 4.67E-06 | -0.01 | 9.97E-01 |
| *GREB1* | 3.9 | 7.78E-05 | 5.1 | 1.18E-07 | 1.2 | 3.22E-01 |
| *RORB* | 3.9 | 5.30E-03 | 6.9 | 3.20E-07 | 3.0 | 4.16E-02 |
| *SCARA5* | 3.8 | 2.32E-04 | 4.3 | 5.48E-06 | 0.6 | 7.03E-01 |
| *CNR1* | 3.7 | 2.86E-04 | 3.3 | 8.76E-04 | -0.4 | 8.27E-01 |
| *NDP* | 3.4 | 1.47E-02 | 6.4 | 4.52E-06 | 3.0 | 4.76E-02 |
| *HLA-DQA1* | 3.3 | 9.10E-03 | 1.6 | 5.58E-01 | -1.7 | 2.41E-01 |
| *RXFP1* | 3.3 | 1.12E-03 | 4.2 | 1.11E-05 | 0.9 | 4.96E-01 |
| *IGFBP6* | 3.2 | 1.56E-03 | 3.9 | 3.99E-05 | 0.8 | 5.70E-01 |
| *PRUNE2* | 3.1 | 3.48E-04 | 4.0 | 4.80E-07 | 0.9 | 3.84E-01 |
| *DKK1* | 3.0 | 5.49E-06 | 3.8 | 7.16E-10 | 0.8 | 3.68E-01 |
| *MEDAG* | 3.0 | 2.20E-03 | 4.1 | 1.11E-05 | 1.1 | 3.77E-01 |
| *LEFTY2* | 3.0 | 3.03E-03 | 2.4 | 2.31E-02 | -0.5 | 7.38E-01 |
| *RGS1* | 2.9 | 7.78E-05 | 2.1 | 6.87E-03 | -0.8 | 3.72E-01 |
| *RASD1* | 2.8 | 2.88E-04 | 2.8 | 2.08E-04 | -0.03 | 9.85E-01 |
| *TRPC4* | 2.8 | 7.44E-04 | 2.4 | 5.42E-03 | -0.4 | 7.45E-01 |
| *ALDH1A1* | 2.7 | 1.87E-05 | 2.8 | 2.75E-06 | 0.1 | 9.54E-01 |
| *RBP4* | 2.7 | 1.46E-02 | 4.3 | 3.79E-05 | 1.6 | 1.76E-01 |
| *DNASE1L3* | 2.7 | 7.44E-04 | 2.3 | 5.18E-03 | -0.4 | 7.73E-01 |
| *NCAM1* | 2.7 | 3.38E-03 | 2.5 | 7.78E-03 | -0.2 | 9.22E-01 |
| *AHNAK2* | 2.6 | 6.43E-04 | 2.9 | 3.34E-05 | 0.3 | 7.92E-01 |
| *CXCL9* | 2.6 | 9.98E-02 | 3.6 | 2.71E-02 | 1.0 | 6.15E-01 |
| *THBS2* | 2.5 | 3.38E-03 | 2.5 | 3.92E-03 | -0.02 | 9.90E-01 |
| *COL4A4* | 2.5 | 3.08E-03 | 2.8 | 4.48E-04 | 0.4 | 7.72E-01 |
| *CP* | 2.4 | 6.08E-03 | 2.2 | 2.20E-02 | -0.3 | 8.57E-01 |
| *WT1* | 2.4 | 1.27E-02 | 3.5 | 1.42E-04 | 1.2 | 3.21E-01 |
| *WDR72* | 2.4 | 1.54E-02 | 2.5 | 1.47E-02 | 0.2 | 9.32E-01 |
| *RAMP1* | 2.3 | 7.19E-04 | 2.4 | 2.61E-04 | 0.1 | 9.26E-01 |
| *ADAMTS4* | 2.3 | 6.48E-04 | 2.6 | 3.99E-05 | 0.3 | 7.79E-01 |
| *PZP* | 2.3 | 2.43E-03 | 1.8 | 2.88E-02 | -0.5 | 6.70E-01 |
| *TMEM132C* | 2.3 | 5.17E-04 | 2.0 | 2.59E-03 | -0.3 | 8.16E-01 |
| *ALPK2* | 2.2 | 1.43E-05 | 1.3 | 2.05E-02 | -0.9 | 1.78E-01 |
| *ALDH1A2* | 2.2 | 3.29E-04 | 1.9 | 1.69E-03 | -0.3 | 7.99E-01 |
| *TNC* | 2.2 | 3.34E-04 | 2.0 | 1.32E-03 | -0.2 | 8.31E-01 |
| *ABI3BP* | 2.2 | 2.88E-04 | 2.1 | 3.29E-04 | -0.1 | 9.37E-01 |
| *AADAC* | 2.2 | 8.72E-03 | 2.4 | 5.61E-03 | 0.2 | 8.93E-01 |
| *C3* | 2.1 | 2.20E-03 | 2.0 | 5.38E-03 | -0.2 | 8.89E-01 |
| *LSAMP* | 2.1 | 1.75E-02 | 2.5 | 6.83E-03 | 0.4 | 8.05E-01 |
| *GAS1* | 2.1 | 2.05E-04 | 2.7 | 2.58E-07 | 0.6 | 4.33E-01 |
| *CXCL10* | 2.1 | 3.48E-02 | 1.6 | 2.86E-01 | -0.5 | 7.49E-01 |
| *IL1RL1* | 2.1 | 1.08E-02 | 1.1 | 5.14E-01 | -0.9 | 3.50E-01 |
| *RGS22* | 2.1 | 2.56E-03 | 1.2 | 2.91E-01 | -0.9 | 3.05E-01 |
| *CFH* | 2.1 | 2.78E-03 | 1.3 | 1.66E-01 | -0.7 | 4.31E-01 |
| *ITK* | 2.0 | 1.26E-02 | 2.1 | 1.19E-02 | 0.1 | 9.70E-01 |
| *PTGIS* | 2.0 | 1.50E-02 | 1.9 | 4.80E-02 | -0.2 | 9.11E-01 |
| *CD96* | 2.0 | 3.00E-04 | 1.1 | 1.22E-01 | -0.9 | 2.16E-01 |
| *CILP* | 2.0 | 9.96E-03 | 2.6 | 3.46E-04 | 0.6 | 5.54E-01 |
| *HSD11B1* | 2.0 | 2.95E-03 | 1.6 | 4.02E-02 | -0.4 | 6.78E-01 |
| *MPZL2* | 2.0 | 1.20E-02 | 2.8 | 2.90E-04 | 0.8 | 4.57E-01 |
| *GBP5* | 2.0 | 1.41E-02 | 2.1 | 1.19E-02 | 0.1 | 9.64E-01 |
| *CD69* | 2.0 | 7.19E-04 | 1.2 | 1.51E-01 | -0.8 | 2.67E-01 |
| *OXTR* | 2.0 | 1.55E-03 | 2.3 | 2.20E-04 | 0.3 | 8.04E-01 |
| *KCND2* | 2.0 | 7.84E-02 | 2.8 | 1.37E-02 | 0.8 | 5.73E-01 |
| *CD38* | 2.0 | 4.31E-03 | 2.4 | 3.29E-04 | 0.4 | 6.89E-01 |
| *NAP1L3* | 1.9 | 2.85E-03 | 1.5 | 4.60E-02 | -0.4 | 6.92E-01 |
| *ATOH8* | 1.9 | 6.93E-04 | 1.6 | 6.73E-03 | -0.3 | 7.33E-01 |
| *CYTIP* | 1.9 | 1.32E-03 | 1.0 | 3.78E-01 | -1.0 | 1.92E-01 |
| *ANOS1* | 1.9 | 3.11E-03 | 1.5 | 4.57E-02 | -0.4 | 6.62E-01 |
| *CHI3L2* | 1.9 | 6.37E-04 | 1.6 | 7.78E-03 | -0.3 | 7.14E-01 |
| *GLB1L2* | 1.9 | 2.21E-02 | 2.9 | 2.20E-04 | 1.0 | 3.15E-01 |
| *CXCL11* | 1.9 | 4.54E-02 | 1.3 | 5.08E-01 | -0.6 | 6.34E-01 |
| *PTGS2* | 1.8 | 5.08E-04 | 1.0 | 2.66E-01 | -0.9 | 1.62E-01 |
| *ZBTB16* | 1.8 | 7.00E-03 | 1.2 | 2.57E-01 | -0.6 | 4.78E-01 |
| *OMD* | 1.8 | 2.56E-03 | 2.2 | 1.61E-04 | 0.4 | 7.01E-01 |
| *ISLR* | 1.8 | 6.34E-04 | 2.0 | 5.43E-05 | 0.2 | 8.19E-01 |
| *SMOX* | 1.8 | 3.66E-03 | 2.4 | 3.25E-05 | 0.6 | 4.65E-01 |
| *TNFRSF11B* | 1.8 | 1.73E-02 | 1.3 | 2.27E-01 | -0.4 | 6.96E-01 |
| *TWIST2* | 1.8 | 1.72E-02 | 1.9 | 1.72E-02 | 0.1 | 9.50E-01 |
| *PRRX1* | 1.8 | 1.08E-02 | 1.2 | 2.75E-01 | -0.6 | 5.44E-01 |
| *GEM* | 1.8 | 1.62E-03 | 1.4 | 2.19E-02 | -0.4 | 6.80E-01 |
| *WISP1* | 1.8 | 7.12E-03 | 1.8 | 7.56E-03 | 0.1 | 9.69E-01 |
| *KCNB1* | 1.8 | 6.08E-03 | 1.8 | 5.61E-03 | 0.1 | 9.63E-01 |
| *ADCY1* | 1.7 | 2.11E-02 | 2.3 | 1.69E-03 | 0.6 | 5.76E-01 |
| *SLC12A8* | 1.7 | 2.13E-02 | 2.1 | 5.61E-03 | 0.4 | 7.43E-01 |
| *PRDM1* | 1.7 | 1.63E-03 | 1.9 | 4.84E-04 | 0.1 | 8.92E-01 |
| *OGN* | 1.7 | 3.65E-03 | 1.7 | 5.42E-03 | -0.03 | 9.82E-01 |
| *DEPTOR* | 1.7 | 3.07E-04 | 1.7 | 4.03E-04 | -0.1 | 9.52E-01 |
| *CTSW* | 1.7 | 2.64E-02 | 1.9 | 1.91E-02 | 0.2 | 8.82E-01 |
| *MOV10L1* | 1.7 | 3.85E-02 | 1.1 | 4.87E-01 | -0.6 | 6.04E-01 |
| *EPDR1* | 1.7 | 1.40E-02 | 2.5 | 1.47E-04 | 0.8 | 3.71E-01 |
| *IL7R* | 1.7 | 2.23E-03 | 1.2 | 9.10E-02 | -0.5 | 5.01E-01 |
| *NR4A2* | 1.7 | 7.01E-04 | 1.1 | 6.23E-02 | -0.6 | 4.09E-01 |
| *IGFBP2* | 1.7 | 9.80E-03 | 1.9 | 3.77E-03 | 0.2 | 8.58E-01 |
| *MAATS1* | 1.7 | 8.42E-03 | 1.7 | 1.94E-02 | -0.02 | 9.85E-01 |
| *CLNK* | 1.7 | 1.76E-02 | 1.5 | 6.71E-02 | -0.2 | 8.96E-01 |
| *PADI1* | 1.7 | 1.93E-01 | 3.1 | 1.12E-02 | 1.4 | 3.36E-01 |
| *ADRA2C* | 1.7 | 1.51E-02 | 1.9 | 7.81E-03 | 0.2 | 8.55E-01 |
| *RBP1* | 1.7 | 6.30E-04 | 1.4 | 4.67E-03 | -0.2 | 7.85E-01 |
| *F2R* | 1.7 | 4.93E-03 | 2.2 | 7.44E-05 | 0.5 | 5.00E-01 |
| *CNTNAP3* | 1.7 | 1.20E-02 | 1.3 | 1.38E-01 | -0.3 | 7.51E-01 |
| *DNER* | 1.6 | 4.24E-02 | 1.3 | 2.80E-01 | -0.3 | 8.11E-01 |
| *MARCO* | 1.6 | 2.90E-02 | 1.0 | 5.88E-01 | -0.7 | 4.87E-01 |
| *TRIM63* | 1.6 | 1.92E-02 | 1.0 | 4.71E-01 | -0.6 | 5.08E-01 |
| *LRRC15* | 1.6 | 1.23E-01 | 2.8 | 7.09E-03 | 1.1 | 3.51E-01 |
| *GGT5* | 1.6 | 1.76E-02 | 1.7 | 1.46E-02 | 0.1 | 9.21E-01 |
| *KLF15* | 1.6 | 1.86E-02 | 1.2 | 2.11E-01 | -0.4 | 7.28E-01 |
| *IL18RAP* | 1.6 | 1.46E-02 | 1.7 | 1.93E-02 | 0.1 | 9.64E-01 |
| *FHOD3* | 1.6 | 2.80E-02 | 2.4 | 3.59E-04 | 0.8 | 3.85E-01 |
| *FNDC4* | 1.6 | 2.93E-03 | 1.7 | 3.92E-03 | 0.1 | 9.64E-01 |
| *IL1R2* | 1.6 | 7.26E-02 | 2.7 | 5.73E-04 | 1.1 | 2.31E-01 |
| *ZNF516* | 1.6 | 1.55E-03 | 1.5 | 2.28E-03 | 0.0 | 9.72E-01 |
| *CPXM1* | 1.6 | 4.09E-03 | 1.6 | 4.67E-03 | 0.005 | 9.96E-01 |
| *SULF1* | 1.6 | 4.39E-03 | 1.2 | 9.48E-02 | -0.4 | 6.10E-01 |
| *C11orf96* | 1.5 | 3.53E-02 | 2.1 | 2.59E-03 | 0.6 | 5.32E-01 |
| *MX2* | 1.5 | 5.08E-04 | 1.1 | 3.76E-02 | -0.5 | 4.53E-01 |
| *SPON2* | 1.5 | 6.60E-03 | 1.6 | 5.18E-03 | 0.1 | 9.32E-01 |
| *KCNQ3* | 1.5 | 3.05E-02 | 1.6 | 3.39E-02 | 0.1 | 9.28E-01 |
| *CD22* | 1.5 | 2.80E-02 | 2.3 | 5.55E-04 | 0.7 | 3.81E-01 |
| *OASL* | 1.5 | 1.08E-02 | 1.2 | 9.67E-02 | -0.3 | 7.65E-01 |
| *PTPN13* | 1.5 | 4.55E-03 | 1.4 | 1.61E-02 | -0.1 | 9.01E-01 |
| *MAOB* | 1.5 | 1.29E-01 | 2.3 | 1.29E-02 | 0.8 | 5.15E-01 |
| *SULF2* | 1.5 | 1.99E-03 | 1.1 | 6.65E-02 | -0.4 | 5.55E-01 |
| *MGST1* | 1.5 | 2.70E-02 | 1.8 | 9.45E-03 | 0.3 | 7.32E-01 |
| *DTNA* | 1.5 | 1.77E-03 | 1.3 | 1.16E-02 | -0.2 | 8.04E-01 |
| *RRAD* | 1.5 | 3.03E-02 | 2.1 | 1.53E-03 | 0.6 | 4.87E-01 |
| *LUM* | 1.4 | 3.54E-03 | 1.5 | 4.67E-03 | 0.004 | 9.97E-01 |
| *WNT9A* | 1.4 | 1.55E-03 | 1.1 | 4.09E-02 | -0.3 | 6.10E-01 |
| *SP140* | 1.4 | 8.90E-03 | 1.0 | 1.96E-01 | -0.4 | 6.11E-01 |
| *SPN* | 1.4 | 4.34E-02 | 1.5 | 5.41E-02 | 0.1 | 9.50E-01 |
| *CCDC141* | 1.4 | 3.67E-02 | 1.5 | 7.83E-02 | 0.047 | 9.73E-01 |
| *RHOH* | 1.4 | 3.69E-02 | 1.3 | 1.34E-01 | -0.1 | 9.01E-01 |
| *COL4A3* | 1.4 | 1.21E-02 | 1.2 | 6.79E-02 | -0.2 | 8.37E-01 |
| *MX1* | 1.4 | 1.50E-03 | 1.1 | 2.25E-02 | -0.3 | 6.84E-01 |
| *IKZF3* | 1.4 | 3.06E-02 | 2.0 | 6.11E-04 | 0.6 | 4.29E-01 |
| *TNFSF10* | 1.4 | 1.92E-02 | 1.2 | 1.24E-01 | -0.2 | 8.32E-01 |
| *SPSB1* | 1.4 | 1.25E-03 | 1.0 | 3.33E-02 | -0.3 | 5.96E-01 |
| *SPHK1* | 1.4 | 3.20E-02 | 2.0 | 8.49E-04 | 0.7 | 4.15E-01 |
| *ITGAX* | 1.3 | 4.76E-02 | 1.2 | 2.13E-01 | -0.1 | 9.07E-01 |
| *KLHL13* | 1.3 | 1.20E-01 | 1.9 | 3.29E-02 | 0.6 | 5.99E-01 |
| *GPR176* | 1.3 | 2.08E-02 | 1.3 | 6.91E-02 | -0.1 | 9.50E-01 |
| *EGR2* | 1.3 | 4.90E-02 | 1.2 | 1.64E-01 | -0.1 | 9.43E-01 |
| *CHST2* | 1.3 | 1.54E-02 | 2.0 | 4.23E-05 | 0.7 | 2.81E-01 |
| *IGSF10* | 1.3 | 2.59E-02 | 1.5 | 8.35E-03 | 0.2 | 7.91E-01 |
| *HMCN2* | 1.3 | 8.37E-02 | 2.1 | 2.28E-03 | 0.9 | 3.06E-01 |
| *FOXO1* | 1.3 | 9.80E-04 | 1.3 | 6.11E-04 | 0.030 | 9.72E-01 |
| *HAND2* | 1.2 | 3.08E-03 | 1.4 | 8.49E-04 | 0.1 | 8.70E-01 |
| *SLAMF6* | 1.2 | 4.41E-02 | 1.3 | 7.18E-02 | 0.041 | 9.74E-01 |
| *MELTF* | 1.2 | 8.04E-03 | 1.5 | 8.49E-04 | 0.3 | 6.91E-01 |
| *COL8A1* | 1.2 | 7.32E-03 | 1.4 | 2.40E-03 | 0.2 | 8.39E-01 |
| *ALDOC* | 1.2 | 4.73E-02 | 1.3 | 8.75E-02 | 0.1 | 9.55E-01 |
| *GPR132* | 1.2 | 1.25E-02 | 1.0 | 7.17E-02 | -0.2 | 8.54E-01 |
| *CD226* | 1.2 | 1.04E-02 | 1.0 | 6.76E-02 | -0.2 | 8.39E-01 |
| *TIPARP* | 1.2 | 7.63E-04 | 1.1 | 4.19E-03 | -0.1 | 8.42E-01 |
| *GPRIN3* | 1.2 | 1.85E-02 | 1.0 | 9.49E-02 | -0.2 | 8.56E-01 |
| *COL12A1* | 1.2 | 9.92E-02 | 1.6 | 3.32E-02 | 0.4 | 6.59E-01 |
| *PLXNA4* | 1.1 | 3.57E-02 | 1.4 | 1.02E-02 | 0.3 | 7.46E-01 |
| *FKBP5* | 1.1 | 6.55E-02 | 1.4 | 2.17E-02 | 0.3 | 7.06E-01 |
| *CHST7* | 1.1 | 1.62E-02 | 1.2 | 1.01E-02 | 0.1 | 8.72E-01 |
| *CD9* | 1.1 | 1.86E-02 | 1.1 | 2.91E-02 | 0.006 | 9.95E-01 |
| *RASAL3* | 1.1 | 3.85E-02 | 1.0 | 1.51E-01 | -0.1 | 9.16E-01 |
| *CTSK* | 1.1 | 4.16E-03 | 1.0 | 1.97E-02 | -0.1 | 8.57E-01 |
| *MAP3K5* | 1.1 | 4.37E-03 | 1.0 | 1.79E-02 | -0.1 | 8.82E-01 |
| *VEGFA* | 1.1 | 4.40E-02 | 1.3 | 1.41E-02 | 0.3 | 7.48E-01 |
| *KCNJ16* | 1.1 | 2.21E-01 | 1.8 | 4.57E-02 | 0.7 | 4.39E-01 |
| *SHC3* | 1.1 | 2.25E-02 | 1.2 | 1.97E-02 | 0.1 | 8.88E-01 |
| *HAPLN3* | 1.1 | 1.34E-01 | 1.7 | 1.72E-02 | 0.6 | 4.84E-01 |
| *C1R* | 1.1 | 2.80E-02 | 1.2 | 1.72E-02 | 0.1 | 8.73E-01 |
| *ART4* | 1.0 | 2.37E-02 | 1.0 | 8.33E-02 | -0.1 | 9.35E-01 |
| *GALM* | 1.0 | 1.54E-02 | 1.1 | 2.57E-02 | 0.012 | 9.90E-01 |
| *SLC43A3* | 1.0 | 6.70E-02 | 1.2 | 4.74E-02 | 0.2 | 8.15E-01 |
| *CRLF1* | 1.0 | 9.43E-02 | 1.3 | 3.92E-02 | 0.3 | 7.08E-01 |
| *PKDCC* | 1.0 | 4.51E-02 | 1.1 | 6.08E-02 | 0.1 | 9.46E-01 |
| *NFASC* | 1.0 | 1.21E-01 | 1.4 | 4.43E-02 | 0.4 | 6.60E-01 |
| *ISG15* | 1.0 | 1.07E-01 | 1.4 | 2.61E-02 | 0.4 | 6.16E-01 |
| *RUNX3* | 1.0 | 2.64E-01 | 1.7 | 4.40E-02 | 0.7 | 4.43E-01 |

*Multiple corrections testing was performed using the Benjamini Hochberg method with a Q value of <0.05.
