# Supplementary material for: Unique transcriptomic landscapes identified in idiopathic spontaneous and infection related preterm births compared to normal term births
Source: PLoS One. 2019 Nov 8;14(11):e0225062. doi: 10.1371/journal.pone.0225062 (PMC6839872; doi:10.1371/journal.pone.0225062)
Supplement: S3 Table — (DOCX) [file pone.0225062.s003.docx]

**S3 Table: Candidate genes associated AHC molecular signature**

|  | **AHC vs TB** | | **AHC vs isPTB** | | **isPTB vs TB** | |
| --- | --- | --- | --- | --- | --- | --- |
| **Gene ID** | **Log2 Fold Change** | **Adjusted**  **P value*** | **Log2 Fold Change** | **Adjusted**  **P value*** | **Log2 Fold Change** | **Adjusted**  **P value*** |
| *CDKN1C* | 3.05 | 1.78E-05 | 3.09 | 4.32E-06 | -0.04 | 9.96E-01 |
| *FIBCD1* | 2.49 | 3.67E-09 | 2.78 | 2.39E-12 | -0.29 | 9.26E-01 |
| *CXCL1* | 2.47 | 4.14E-05 | 2.14 | 2.83E-04 | 0.33 | 9.29E-01 |
| *NXPH4* | 2.12 | 7.25E-07 | 2.02 | 7.00E-07 | 0.10 | 9.75E-01 |
| *WNT7B* | 1.85 | 1.07E-04 | 1.35 | 4.67E-03 | 0.50 | 8.49E-01 |
| *BANF1* | 1.80 | 8.36E-12 | 2.01 | 4.26E-16 | -0.21 | 8.95E-01 |
| *RRM2* | 1.66 | 7.87E-07 | 1.36 | 3.76E-05 | 0.31 | 8.83E-01 |
| *CRCT1* | 1.66 | 5.50E-02 | 1.68 | 3.86E-02 | -0.02 | 9.98E-01 |
| *NPIPB4* | 1.62 | 2.82E-02 | 1.43 | 4.07E-02 | 0.32 | 9.15E-01 |
| *REEP6* | 1.62 | 1.45E-02 | 1.35 | 3.55E-02 | 0.27 | 9.52E-01 |
| *MYO1A* | 1.61 | 9.16E-03 | 1.10 | 8.00E-02 | 0.51 | 8.84E-01 |
| *RBPMS2* | 1.57 | 1.69E-03 | 1.17 | 1.96E-02 | 0.39 | 8.88E-01 |
| *H2AFX* | 1.50 | 8.36E-12 | 1.50 | 9.72E-13 | 0.00 | 9.99E-01 |
| *KIF18B* | 1.50 | 6.08E-06 | 1.31 | 4.69E-05 | 0.18 | 9.37E-01 |
| *RPS16* | 1.49 | 1.13E-06 | 1.38 | 3.90E-06 | 0.11 | 9.67E-01 |
| *HMBS* | 1.48 | 2.60E-06 | 1.31 | 1.87E-05 | 0.17 | 9.42E-01 |
| *ZSWIM9* | 1.48 | 2.81E-05 | 1.18 | 6.67E-04 | 0.30 | 8.85E-01 |
| *SAPCD2* | 1.46 | 3.44E-04 | 1.18 | 3.22E-03 | 0.28 | 9.11E-01 |
| *SRM* | 1.44 | 2.38E-06 | 1.16 | 1.05E-04 | 0.28 | 8.77E-01 |
| *CXorf67* | 1.42 | 2.06E-05 | 1.27 | 1.27E-04 | 0.15 | 9.52E-01 |
| *FOXM1* | 1.41 | 4.04E-07 | 1.16 | 1.85E-05 | 0.25 | 8.85E-01 |
| *SUSD2* | 1.41 | 1.04E-05 | 1.62 | 5.55E-08 | -0.21 | 9.13E-01 |
| *FAM83F* | 1.39 | 3.52E-02 | 0.73 | 2.96E-01 | 0.67 | 8.16E-01 |
| *PHOSPHO1* | 1.37 | 2.20E-03 | 1.02 | 1.92E-02 | 0.34 | 8.85E-01 |
| *PPDPF* | 1.36 | 2.38E-03 | 1.00 | 2.75E-02 | 0.36 | 8.85E-01 |
| *HIST1H4A* | 1.34 | 1.98E-04 | 1.44 | 1.87E-05 | -0.10 | 9.72E-01 |
| *DLX4* | 1.34 | 2.44E-05 | 1.15 | 3.08E-04 | 0.19 | 9.27E-01 |
| *PTMS* | 1.32 | 2.81E-06 | 1.12 | 4.06E-05 | 0.19 | 9.09E-01 |
| *CENPA* | 1.31 | 1.04E-03 | 1.01 | 1.04E-02 | 0.30 | 8.90E-01 |
| *TYMS* | 1.30 | 9.28E-08 | 1.33 | 9.88E-09 | -0.02 | 9.91E-01 |
| *PPP1R14A* | 1.30 | 2.97E-02 | 1.18 | 4.07E-02 | 0.12 | 9.75E-01 |
| *CDCA5* | 1.30 | 2.89E-05 | 1.41 | 1.91E-06 | -0.10 | 9.67E-01 |
| *HIST1H2BM* | 1.28 | 5.33E-03 | 0.96 | 3.67E-02 | 0.32 | 8.90E-01 |
| *GTSE1* | 1.28 | 1.57E-05 | 1.27 | 6.98E-06 | 0.00 | 9.99E-01 |
| *TK1* | 1.26 | 6.77E-06 | 1.33 | 5.76E-07 | -0.07 | 9.75E-01 |
| *CENPM* | 1.24 | 1.97E-03 | 1.40 | 2.39E-04 | -0.15 | 9.63E-01 |
| *HMGA1* | 1.24 | 7.07E-05 | 1.26 | 2.12E-05 | -0.02 | 9.95E-01 |
| *SHCBP1* | 1.23 | 4.82E-05 | 1.23 | 1.87E-05 | -0.01 | 9.99E-01 |
| *CDC20* | 1.22 | 9.18E-05 | 1.10 | 3.28E-04 | 0.12 | 9.63E-01 |
| *CDT1* | 1.22 | 4.75E-06 | 1.27 | 5.62E-07 | -0.06 | 9.78E-01 |
| *E2F7* | 1.21 | 1.01E-04 | 1.01 | 9.26E-04 | 0.20 | 9.24E-01 |
| *TICRR* | 1.20 | 1.16E-05 | 1.12 | 2.07E-05 | 0.08 | 9.74E-01 |
| *ESPL1* | 1.20 | 1.69E-04 | 0.86 | 8.04E-03 | 0.34 | 8.38E-01 |
| *UHRF1* | 1.19 | 3.52E-03 | 0.90 | 2.72E-02 | 0.30 | 8.90E-01 |
| *IGF2* | 1.19 | 5.09E-03 | 0.79 | 7.52E-02 | 0.40 | 8.59E-01 |
| *MAGEA4* | 1.18 | 1.05E-01 | 1.85 | 7.47E-03 | -0.67 | 8.49E-01 |
| *FAM111B* | 1.18 | 2.71E-02 | 0.86 | 1.02E-01 | 0.32 | 9.11E-01 |
| *RNF26* | 1.17 | 3.00E-09 | 1.24 | 3.58E-11 | -0.06 | 9.71E-01 |
| *TICAM1* | 1.17 | 8.92E-06 | 1.00 | 1.29E-04 | 0.17 | 9.21E-01 |
| *CDKN2A* | 1.17 | 2.16E-03 | 0.88 | 2.14E-02 | 0.29 | 8.90E-01 |
| *QDPR* | 1.17 | 8.91E-04 | 0.93 | 8.19E-03 | 0.24 | 9.00E-01 |
| *PCLAF* | 1.17 | 2.00E-02 | 1.37 | 2.68E-03 | -0.20 | 9.52E-01 |
| *PSAT1* | 1.17 | 1.16E-02 | 1.10 | 1.28E-02 | 0.07 | 9.85E-01 |
| *MYBL2* | 1.15 | 4.48E-05 | 1.19 | 1.32E-05 | -0.03 | 9.88E-01 |
| *PLK1* | 1.15 | 1.48E-06 | 1.11 | 1.70E-06 | 0.04 | 9.81E-01 |
| *CDC25A* | 1.15 | 2.26E-04 | 1.28 | 1.55E-05 | -0.13 | 9.55E-01 |
| *HIST1H3G* | 1.15 | 5.65E-04 | 1.07 | 8.03E-04 | 0.08 | 9.75E-01 |
| *PRR11* | 1.13 | 1.02E-04 | 1.02 | 3.28E-04 | 0.11 | 9.63E-01 |
| *FAM83D* | 1.13 | 2.67E-04 | 1.07 | 3.40E-04 | 0.06 | 9.80E-01 |
| *MKI67* | 1.13 | 9.23E-05 | 1.00 | 4.23E-04 | 0.13 | 9.50E-01 |
| *HRCT1* | 1.13 | 3.96E-02 | 0.98 | 6.19E-02 | 0.15 | 9.72E-01 |
| *HIST1H3H* | 1.13 | 7.89E-04 | 0.89 | 7.56E-03 | 0.23 | 8.94E-01 |
| *BIRC5* | 1.12 | 2.05E-03 | 1.24 | 2.95E-04 | -0.11 | 9.67E-01 |
| *FJX1* | 1.12 | 8.14E-03 | 0.98 | 1.79E-02 | 0.15 | 9.63E-01 |
| *HIST1H3I* | 1.12 | 1.36E-03 | 0.90 | 1.00E-02 | 0.23 | 9.11E-01 |
| *KLF16* | 1.12 | 1.16E-05 | 0.89 | 5.00E-04 | 0.23 | 8.84E-01 |
| *WNK2* | 1.12 | 1.12E-03 | 0.68 | 6.01E-02 | 0.44 | 6.95E-01 |
| *ATN1* | 1.12 | 4.94E-04 | 0.93 | 3.60E-03 | 0.19 | 9.27E-01 |
| *SLC25A29* | 1.12 | 1.94E-03 | 0.75 | 4.36E-02 | 0.37 | 8.36E-01 |
| *CNOT3* | 1.11 | 4.87E-02 | 0.92 | 8.65E-02 | 0.19 | 9.63E-01 |
| *FOXI3* | 1.11 | 9.74E-04 | 1.07 | 8.41E-04 | 0.04 | 9.88E-01 |
| *HIST1H2AL* | 1.10 | 1.86E-03 | 1.06 | 1.67E-03 | 0.04 | 9.88E-01 |
| *CDH3* | 1.10 | 2.26E-03 | 1.01 | 3.97E-03 | 0.09 | 9.75E-01 |
| *SEPHS2* | 1.10 | 5.34E-08 | 1.13 | 5.39E-09 | -0.03 | 9.88E-01 |
| *SGO1* | 1.10 | 4.78E-03 | 0.92 | 1.54E-02 | 0.18 | 9.47E-01 |
| *ILDR1* | 1.10 | 6.79E-03 | 1.06 | 5.23E-03 | 0.03 | 9.93E-01 |
| *FAM167B* | 1.09 | 4.21E-02 | 1.07 | 2.91E-02 | 0.02 | 9.98E-01 |
| *AURKB* | 1.08 | 1.16E-03 | 1.11 | 4.65E-04 | -0.03 | 9.93E-01 |
| *PMVK* | 1.08 | 2.96E-04 | 1.18 | 2.23E-05 | -0.10 | 9.67E-01 |
| *BICRA* | 1.08 | 4.15E-04 | 0.87 | 4.78E-03 | 0.21 | 8.97E-01 |
| *TPX2* | 1.08 | 1.83E-04 | 1.05 | 1.61E-04 | 0.03 | 9.88E-01 |
| *CCNF* | 1.08 | 1.45E-05 | 0.87 | 4.21E-04 | 0.20 | 8.88E-01 |
| *RAD51* | 1.07 | 4.75E-06 | 1.22 | 3.28E-08 | -0.14 | 9.27E-01 |
| *SP6* | 1.07 | 3.18E-04 | 1.21 | 1.68E-05 | -0.14 | 9.48E-01 |
| *KIF4A* | 1.07 | 1.87E-03 | 0.90 | 7.12E-03 | 0.16 | 9.47E-01 |
| *TP53I11* | 1.06 | 8.93E-04 | 0.64 | 5.92E-02 | 0.43 | 6.67E-01 |
| *PKMYT1* | 1.06 | 1.32E-03 | 1.01 | 1.48E-03 | 0.05 | 9.85E-01 |
| *GET4* | 1.06 | 3.10E-02 | 0.78 | 1.13E-01 | 0.28 | 9.16E-01 |
| *CLIP3* | 1.06 | 2.38E-02 | 0.79 | 8.82E-02 | 0.27 | 9.18E-01 |
| *LRFN3* | 1.06 | 1.87E-02 | 0.63 | 1.81E-01 | 0.43 | 8.49E-01 |
| *CDCP1* | 1.05 | 2.97E-02 | 0.67 | 1.80E-01 | 0.38 | 8.75E-01 |
| *CLSPN* | 1.05 | 3.59E-03 | 1.11 | 8.68E-04 | -0.06 | 9.80E-01 |
| *RAB11B* | 1.04 | 5.84E-06 | 1.03 | 4.45E-06 | 0.01 | 9.95E-01 |
| *KRT10* | 1.04 | 5.15E-04 | 0.90 | 2.39E-03 | 0.13 | 9.51E-01 |
| *CMBL* | 1.04 | 5.33E-03 | 0.88 | 1.43E-02 | 0.15 | 9.52E-01 |
| *ADAMTS19* | 1.04 | 6.21E-03 | 1.03 | 3.42E-03 | 0.01 | 9.98E-01 |
| *HIST1H1B* | 1.04 | 1.12E-03 | 0.93 | 2.62E-03 | 0.11 | 9.63E-01 |
| *CDCA2* | 1.03 | 4.58E-03 | 1.11 | 1.12E-03 | -0.08 | 9.77E-01 |
| *RTL8A* | 1.03 | 1.77E-04 | 1.07 | 4.69E-05 | -0.04 | 9.86E-01 |
| *SLC1A2* | 1.03 | 3.17E-02 | 0.66 | 1.74E-01 | 0.37 | 8.80E-01 |
| *HPRT1* | 1.02 | 2.45E-04 | 0.94 | 5.52E-04 | 0.08 | 9.74E-01 |
| *MT2A* | 1.02 | 2.20E-03 | 0.71 | 3.88E-02 | 0.31 | 8.60E-01 |
| *SOX14* | 1.02 | 2.98E-03 | 1.00 | 2.25E-03 | 0.02 | 9.96E-01 |
| *ZNF865* | 1.02 | 1.12E-03 | 0.84 | 6.56E-03 | 0.17 | 9.29E-01 |
| *FOXP4* | 1.01 | 5.09E-05 | 0.70 | 6.64E-03 | 0.31 | 7.54E-01 |
| *CDC45* | 1.01 | 6.12E-03 | 0.87 | 1.51E-02 | 0.14 | 9.59E-01 |
| *LMNB1* | 1.01 | 8.51E-04 | 0.93 | 1.39E-03 | 0.08 | 9.75E-01 |
| *CCDC86* | 1.01 | 3.78E-03 | 0.80 | 2.03E-02 | 0.21 | 9.25E-01 |
| *CKAP2L* | 1.00 | 5.15E-04 | 0.91 | 1.12E-03 | 0.09 | 9.67E-01 |
| *ZNF703* | 1.00 | 1.26E-02 | 1.20 | 1.18E-03 | -0.19 | 9.36E-01 |
| *IGDCC3* | 1.00 | 8.91E-04 | 1.08 | 1.29E-04 | -0.08 | 9.72E-01 |
| *POLR3K* | 0.98 | 7.83E-04 | 1.02 | 2.30E-04 | -0.04 | 9.86E-01 |
| *CCNA2* | 0.97 | 5.44E-04 | 1.14 | 1.32E-05 | -0.17 | 9.16E-01 |
| *UBE2C* | 0.97 | 2.78E-03 | 1.24 | 2.61E-05 | -0.27 | 8.84E-01 |
| *ASF1B* | 0.96 | 2.16E-03 | 1.08 | 2.81E-04 | -0.11 | 9.63E-01 |
| *LYPD3* | 0.96 | 1.26E-02 | 1.08 | 2.56E-03 | -0.12 | 9.67E-01 |
| *SKA3* | 0.94 | 4.38E-03 | 1.11 | 2.93E-04 | -0.17 | 9.33E-01 |
| *ANP32B* | 0.93 | 1.32E-06 | 1.05 | 5.39E-09 | -0.12 | 9.26E-01 |
| *PTTG1* | 0.92 | 6.53E-03 | 1.10 | 4.00E-04 | -0.18 | 9.29E-01 |
| *CENPW* | 0.91 | 3.31E-02 | 1.02 | 9.14E-03 | -0.11 | 9.74E-01 |
| *AP1S3* | 0.89 | 7.58E-03 | 1.03 | 7.83E-04 | -0.14 | 9.51E-01 |
| *PBK* | 0.88 | 3.10E-02 | 1.03 | 5.25E-03 | -0.16 | 9.52E-01 |
| *NETO2* | 0.87 | 3.01E-02 | 1.00 | 5.87E-03 | -0.13 | 9.63E-01 |
| *SMAGP* | 0.87 | 2.05E-03 | 1.04 | 7.46E-05 | -0.16 | 9.25E-01 |
| *AP3B2* | 0.86 | 1.43E-01 | 1.30 | 1.33E-02 | -0.43 | 8.84E-01 |
| *LARGE2* | 0.83 | 1.83E-02 | 1.09 | 4.62E-04 | -0.27 | 8.85E-01 |
| *RPS21* | 0.83 | 1.30E-03 | 1.02 | 1.87E-05 | -0.19 | 8.90E-01 |
| *FZD10* | 0.80 | 1.25E-01 | 1.05 | 2.19E-02 | -0.25 | 9.27E-01 |
| *CDCA8* | 0.80 | 7.96E-03 | 1.07 | 9.58E-05 | -0.27 | 8.61E-01 |
| *DUSP9* | 0.77 | 8.79E-02 | 1.04 | 7.74E-03 | -0.27 | 8.95E-01 |
| *FBXO24* | 0.74 | 1.78E-01 | 1.03 | 3.14E-02 | -0.30 | 9.21E-01 |
| *BCAM* | 0.71 | 1.54E-02 | 1.02 | 1.11E-04 | -0.30 | 8.04E-01 |
| *LAMA1* | 0.69 | 1.67E-01 | 1.12 | 7.12E-03 | -0.43 | 8.42E-01 |
| *CPS1* | 0.67 | 1.94E-01 | 1.04 | 1.65E-02 | -0.37 | 8.68E-01 |
| *CLCA2* | 0.66 | 2.76E-01 | 1.02 | 4.70E-02 | -0.36 | 8.90E-01 |
| *KRTAP26-1* | 0.50 | 4.49E-01 | 1.13 | 3.59E-02 | -0.62 | 7.14E-01 |
| *HTR1D* | 0.49 | 3.89E-01 | 1.02 | 2.52E-02 | -0.53 | 7.55E-01 |
| *CFAP54* | -0.96 | 1.02E-01 | -1.32 | 1.01E-02 | 0.36 | 8.90E-01 |
| *PKD1L1* | -0.97 | 5.63E-02 | -1.03 | 2.97E-02 | 0.06 | 9.87E-01 |
| *MPP3* | -0.98 | 6.45E-02 | -1.07 | 2.73E-02 | 0.10 | 9.76E-01 |
| *MEGF10* | -1.01 | 3.67E-02 | -1.15 | 9.55E-03 | 0.14 | 9.67E-01 |
| *DAPP1* | -1.02 | 1.92E-02 | -0.87 | 4.18E-02 | -0.14 | 9.63E-01 |
| *FGL1* | -1.03 | 4.67E-02 | -1.00 | 3.93E-02 | -0.02 | 9.96E-01 |
| *SSBP1* | -1.03 | 7.63E-04 | -0.70 | 2.75E-02 | -0.33 | 8.00E-01 |
| *AMY2B* | -1.03 | 1.53E-03 | -0.97 | 2.24E-03 | -0.06 | 9.79E-01 |
| *GBGT1* | -1.04 | 4.66E-03 | -0.84 | 2.11E-02 | -0.20 | 9.26E-01 |
| *CFAP58* | -1.04 | 1.13E-02 | -0.90 | 2.90E-02 | -0.14 | 9.62E-01 |
| *PILRA* | -1.05 | 6.84E-02 | -1.47 | 4.06E-03 | 0.42 | 8.77E-01 |
| *MYBPHL* | -1.07 | 4.30E-02 | -0.90 | 9.10E-02 | -0.17 | 9.63E-01 |
| *LAMB4* | -1.08 | 1.18E-02 | -0.96 | 2.40E-02 | -0.12 | 9.67E-01 |
| *C11orf52* | -1.08 | 3.97E-02 | -0.77 | 1.55E-01 | -0.31 | 9.00E-01 |
| *C2CD6* | -1.09 | 4.33E-02 | -0.93 | 8.07E-02 | -0.16 | 9.63E-01 |
| *GALNT15* | -1.12 | 1.05E-01 | -1.52 | 1.27E-02 | 0.40 | 8.94E-01 |
| *CASQ1* | -1.12 | 2.64E-01 | -2.00 | 2.18E-02 | 0.88 | 7.92E-01 |
| *XCL1* | -1.14 | 8.41E-03 | -0.99 | 2.19E-02 | -0.15 | 9.58E-01 |
| *FCN1* | -1.17 | 2.35E-02 | -0.89 | 8.10E-02 | -0.27 | 9.21E-01 |
| *WDR49* | -1.17 | 8.46E-03 | -1.05 | 1.69E-02 | -0.12 | 9.67E-01 |
| *DDI1* | -1.30 | 6.46E-02 | -1.69 | 7.89E-03 | 0.39 | 9.10E-01 |
| *ANG* | -1.41 | 2.97E-02 | -1.47 | 1.56E-02 | 0.06 | 9.88E-01 |
| *ZNF593* | -1.45 | 1.61E-02 | -1.30 | 2.72E-02 | -0.15 | 9.71E-01 |
| *ABCA9* | -1.51 | 1.22E-04 | -1.59 | 2.52E-05 | 0.09 | 9.75E-01 |
| *HBA2* | -1.56 | 2.96E-02 | -1.57 | 1.73E-02 | 0.01 | 9.99E-01 |
| *GAPT* | -1.57 | 2.05E-02 | -1.43 | 2.99E-02 | -0.14 | 9.75E-01 |
| *PPP1R2B* | -1.59 | 1.45E-05 | -1.39 | 2.03E-04 | -0.20 | 9.27E-01 |
| *ABCA6* | -1.62 | 1.03E-04 | -1.86 | 3.94E-06 | 0.24 | 9.26E-01 |
| *HBB* | -1.80 | 3.99E-03 | -1.56 | 9.14E-03 | -0.24 | 9.52E-01 |
| *ADH1B* | -1.93 | 5.82E-03 | -1.73 | 1.09E-02 | -0.20 | 9.67E-01 |
| *ACSM5* | -2.04 | 5.15E-04 | -2.45 | 6.26E-06 | 0.41 | 8.85E-01 |
| *MAP1LC3C* | -2.25 | 1.79E-04 | -2.67 | 5.03E-06 | 0.42 | 8.90E-01 |
| *ADAMDEC1* | -2.94 | 1.97E-04 | -3.31 | 1.68E-05 | 0.36 | 9.39E-01 |

* Multiple corrections testing was performed using the Benjamini Hochberg method with a Q value of <0.05.
